# Utility of a prognostic assessment tool to predict survival following surgery for brain metastases

**DOI:** 10.1093/nop/npad047

**Published:** 2023-08-07

**Authors:** Hadleigh Cuthbert, Max Riley, Shreya Bhatt, Claudia Kate Au-Yeung, Ayesha Arshad, Sondos Eladawi, Athanasios Zisakis, Georgios Tsermoulas, Colin Watts, Victoria Wykes

**Affiliations:** Department of Neurosurgery, Queen Elizabeth Hospital, Mindelsohn Way, Birmingham, UK; University of Birmingham Medical School, Birmingham, UK; University of Birmingham Medical School, Birmingham, UK; University of Warwick Medical School, Coventry, UK; Department of Neurosurgery, Queen Elizabeth Hospital, Mindelsohn Way, Birmingham, UK; Department of Neurosurgery, Queen Elizabeth Hospital, Mindelsohn Way, Birmingham, UK; Department of Neurosurgery, Queen Elizabeth Hospital, Mindelsohn Way, Birmingham, UK; Department of Neurosurgery, Queen Elizabeth Hospital, Mindelsohn Way, Birmingham, UK; Department of Neurosurgery, Queen Elizabeth Hospital, Mindelsohn Way, Birmingham, UK; Institute of Cancer and Genomic Sciences, University of Birmingham, Birmingham, UK; Department of Neurosurgery, Queen Elizabeth Hospital, Mindelsohn Way, Birmingham, UK; Institute of Cancer and Genomic Sciences, University of Birmingham, Birmingham, UK

**Keywords:** brain metastases, graded prognostic assessment, outcome, prognosis, resection

## Abstract

**Background:**

Brain metastases account for more than 50% of all intracranial tumors and are associated with poor outcomes. Treatment decisions in this highly heterogenous cohort remain controversial due to the myriad of treatment options available, and there is no clearly defined standard of care. The prognosis in brain metastasis patients varies widely with tumor type, extracranial disease burden and patient performance status. Decision-making regarding treatment is, therefore, tailored to each patient and their disease.

**Methods:**

This is a retrospective cohort study assessing survival outcomes following surgery for brain metastases over a 50-month period (April 1, 2014–June 30, 2018). We compared predicted survival using the diagnosis-specific Graded Prognostic Assessment (ds-GPA) with actual survival.

**Results:**

A total of 186 patients were included in our cohort. Regression analysis demonstrated no significant correlation between actual and predicted outcome. The most common reason for exclusion was insufficient information being available to the neuro-oncology multidisciplinary team (MDT) meeting to allow GPA calculation.

**Conclusions:**

In this study, we demonstrate that “predicted survival” using the ds-GPA does not correlate with “actual survival” in our operated patient cohort. We also identify a shortcoming in the amount of information available at MDT in order to implement the GPA appropriately. Patient selection for aggressive therapies is crucial, and this study emphasizes the need for treatment decisions to be individualized based on patient and cancer clinical characteristics.

Brain metastases (BMs) affect 40% of patients with extracranial primary cancers, making them the most common intracranial tumors. An estimated 16,000 patients are diagnosed with a brain metastasis annually in the UK, and this burden is expected to grow.^1,2^ An increased emphasis on screening brain imaging for at-risk patients with metastatic cancer,^3^ coupled with improved surveillance and systemic disease control, will contribute to an increased burden of this disease.^4–6^ The impact of BMs is significant for both patients and their families, having major effects on survival, and both cognitive and physical capabilities.

Currently available treatment options for brain metastases include surgery, radiotherapy, and systemic pharmacotherapies. Treatment decisions remain controversial due to the heterogeneity of this patient cohort. Surgery has an important role to play in both tumor resection and for tissue diagnosis in cases where the primary cancer is unknown. The aim of surgical resection is maximum safe resection of the tumor, and this carries the advantages of: reducing mass effect, reducing dependence on steroids, and improving progression-free and overall survival with preservation of functional capacity.^7^ Radiation therapy is recommended for all patients following resection of brain metastases and improves intracranial control and survival outcomes.^8^

However, there are additional treatment options available for BMs besides surgery. SRS is of benefit in multiple or smaller lesions, surgically inaccessible lesions, or poor surgical candidates who may not be able to tolerate an operation or general anesthetic.^9–11^ While localized treatments like surgery and radiotherapy remain the mainstay of treatment, there are an increasing number of targeted systemic therapies and immunotherapies now available. Treatments targeted against specific molecular alterations such as EGFR-mutant and ALK-mutant non-small cell lung cancer, BRAF-mutant melanoma, and HER2-positive breast cancer have led to substantially improved outcomes in these patients,^12^ some of whom would previously face the most dismal outcomes. Immunotherapies are widely used in melanoma, and accordingly patterns of survival in advanced melanoma have improved significantly, since the introduction of such treatments.^13^ The potential efficacy of immunotherapies in other primary tumors, and possible combinations of targeted and local therapies, represent an exciting new frontier in management of BMs.

Despite advances in treatment, outcomes remain poor due to presentation often representing end-stage disease as well as due to the morbidity associated with the BMs themselves. The median survival time of patients undergoing primary surgery for BMs is 10 months.^14^ Not only has this prevented the involvement of BM patients in clinical trials, for fear of masking therapeutic benefits of the treatments under investigation,^15^ but it has also hindered quality prospective studies from being undertaken—from which accurate predictive tools, able to determine outcome after treatment, could be derived.^16,17^

Surgical resection for BMs carries considerable morbidity (3.9%–6%) and mortality (0.7%–1.9%).^18^ The BM cohort is highly heterogenous, and surgical intervention achieves better outcomes in some subgroups of patients than others.^19^ It is, therefore, crucial to identify patients with a better prognosis who may be candidates for more aggressive therapy. There is no absolute cutoff in terms of life expectancy for suitable candidates, but it is generally agreed a performance status of 0–2, and a prognosis of at least 6 months is necessary to assist surgical selection.

Ultimately, surgical treatment recommendations are made at the multidisciplinary team (MDT) level, prior to clinical review and informed decision-making with the patient and care-givers. Prognostic factors such as age, tumor characteristics, number of brain metastases, burden of extracranial primary disease, and performance status all play an important role in guiding individualized treatment options.^20^ The obstacles posed by the heterogeneity of the patient population and the wide range of outcomes have prompted the creation of prognostic tools, intended to be used to predict survival time and facilitate multidisciplinary decision-making.

Recursive Partitioning Analysis (RPA) employed by the Radiation Therapy Oncology Group (RTOG) on BM trials was the first of these tools applied to decision-making.^21^ It sought to stratify the cohort into 3 classes, with aggressive surgical intervention being reserved only for RPA Class I patients who seemed to be most likely to benefit from aggressive treatment strategies.^22^ It was not until 2008 that the Graded Prognostic Assessment (GPA) index was created, to provide a more quantitative and easier-to-use tool.^23^ The GPA also included another validated prognosticator—the number of brain metastases.

It is increasingly evident that primary tumor origin and specific tumor molecular profiles also significantly influence prognosis,^24^ and that prognostic factors vary according to the primary tumor type. A diagnosis-specific GPA (ds-GPA) index was thus introduced which factored in the primary cancer and molecular subtypes. This index has been robustly validated in the years, since its introduction^20,25^ and has benefits in guiding individualized treatment options.

## Materials and Methods

The patient cohort was identified retrospectively from a search of the Department of Pathology database between April 1, 2014 and June 30, 2018 for a confirmed diagnosis of BMs defined as any secondary metastasis to the brain from a solid organ extracranial primary. The primary endpoint for analysis was the survival time from the date of their initial first neurosurgical resection for a brain metastasis. We then reviewed electronic and imaging records to retrospectively calculate a ds-GPA for each patient based on information that will have been available to the operating team before the time of the initial operation. The ds-GPA was used to calculate a “predicted survival” time for each patient.

Only patients undergoing surgery for the purpose of resection of a confirmed and newly diagnosed brain metastasis were included. Exclusion criteria included: primary brain tumors, patients initially managed nonsurgically, biopsy-only, and where insufficient information was available to calculate a ds-GPA. Patients were excluded if any component of the ds-GPA score was missing as this provided insufficient information to calculate a score. In those patients who passed away before 6 months, medical notes were reviewed to establish a cause of death as “neurological” vs. “non-neurological.” Non-neurological was defined as extracranial disease progression or intercurrent systemic illness, such as pneumonia or pulmonary embolus.

Descriptive analysis was completed using Microsoft Office Excel. Statistical analysis was imported to IBM SPSS Statistics 27 for analysis. Multinomial regression analysis was performed to examine statistical correlation between actual and predicted survival years. “Predicted survival” years was set as the independent variable and “actual/observed survival” years as the dependent variable. Significance was set at *P* < .05.

To further analyze the strength of predictive value among different factors, patients were stratified according to actual survival years and primary cancer origin of the metastasis. In regard to actual survival years, 4 subgroups were analyzed: (1) patients with <6 months survival, (2) 6–12 months survival, (3) 1–2-year survival, and (4) >2-year survival. The metastasis subgroups were recorded as per the primary solid organ cancer: breast, lung melanoma, colorectal, renal, etc.

### Ethics Approval and Consent to Participate

This study was registered as a qualitative improvement study at the Queen Elizabeth Hospital Birmingham Research and Audit Department (Registration Number CARMS-16861), and no ethical approval was required.

## Results

**Figure 1.**
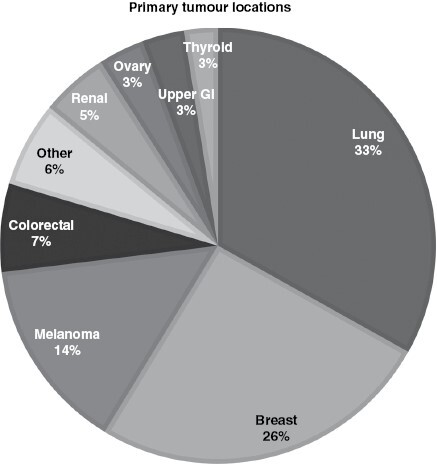
Etiologies of primary cancer locations of patients with brain metastasis in our overall unselected surgical BM cohort.

A total of 186/312 (59%) of reviewed patients were identified to meet inclusion criteria for analysis. The median age of this cohort was 60 years (range 27–82 years), with a 3:2 female predominance. The mean follow-up time was 15.2 months (4 days–85 months). The most common reasons for exclusion were due to insufficient data available (51%, *n* = 64), no GPA for their primary tumor type (24%, *n* = 31), and unknown primary site at time of operation (13%, *n* = 16). Other reasons for exclusion were included in Table 1. There was a wide range of primary cancer locations in the overall unselected surgical BM cohort, with the most common primaries being lung (33%), breast (26%), and melanoma (14%) (see Figure 1). The breakdown of ds-GPA score for each tissue diagnosis of tumor in our included patients can be seen in Table 2.

**Table 1.** Reasons of Exclusion for Analysis

Reason of exclusion	% of cohort
Insufficient information	51
Primary unknown	13
No GPA available for primary tumour type	24
Extracranial primary confirmed postoperatively	3
Emergency admission	2
Recurrent tumour	2
Outside date range	2
Diagnostic uncertainty	1
Unknown reason	2

**Table 2.** Breakdown of ds-GPA Index Scores for Each Tissue Diagnosis of Tumor to Reflect the Distribution of Patients With Different Index Scores.

Primary tumor location	*n*	Disease-specific GPA score
Breast (*n* = 51)	6	1
	9	1.5
	11	2
	6	2.5
	11	3
	6	3.5
	2	4
Gastrointestinal (*n* = 26)	1	0.5
	3	1
	6	1.5
	2	2
	5	2.5
	2	3
	7	3.5
Lung (*n* = 68)	6	1
	13	1.5
	16	2
	10	2.5
	20	3
	1	3.5
	2	4
Melanoma (*n* = 31)	2	0.5
	2	1
	3	1.5
	9	2
	6	2.5
	6	3
	1	3.5
	2	4
Renal cell carcinoma (*n* = 10)	1	0.5
	0	1
	2	1.5
	1	2
	2	2.5
	1	3
	2	3.5
	1	4

**Figure 2.**
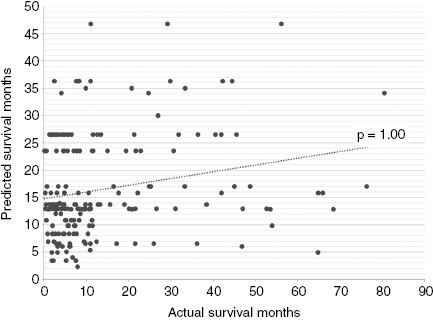
Multinominal regression analysis between actual and predicted survival of patients following first surgery from brain metastasis calculated using the diagnosis-specific Graded Prognostic Assessment (ds-GPA) in the overall cohort. The ds-GPA did not significant predict “actual” survival years (*P* = 1.00).

Multinominal regression analysis revealed that the “predicted survival” years calculated by the ds-GPA did not significantly predict the “actual survival” years (*R*^2^ = 0.07, *P* = 1.00). See Figure 2 for details. Further analyses compared the predictability power of ds-GPA with BM subgroups of primary solid organ cancer location and survival time. There was no significant predictability in patients who survived less than 6 months (*R*^2^ = <0.001, *P* = 1.00), 6–12 months (*R*^2^ = 0.05, *P* = .71), 12–24 months (*R*^2^ = 0.01, *P* = .99), and more than 24 months (*R*^2^ = 0.02, *P* = .98). Furthermore, there was no significant predictability among all subgroups of primary tumor locations.

## Discussion

Our patient cohort clearly highlights the challenges faced in the management of BMs. The cohort is highly heterogenous, with over 20 different primary tumor types included. The demographics of the patients also varies widely. Given that both primary tumor type and patient factors carry prognostic significance, this is emblematic of the problem faced by clinicians—where there is no “one size fits all” treatment approach that can be applied to the management of BMs.

Despite medical advancements, the outcomes following treatment for brain metastases remain poor. We report a median overall survival of 8.1 months, which is slightly lower than published literature.^19,26–29^ Strikingly, early mortality was significant, with 39.6% of patients surviving for less than 6 months after their initial BM diagnosis. When considering cause of death in these patients, the majority were non-neurological in nature. The surgical management of brain metastasis is invasive and demanding on patients and their families, especially when considering time for rehabilitation and recovery following the operation. The fact that many patients who underwent surgery who passed away early did so from non-neurosurgical causes suggests that surgery may not have been appropriate for these patients in the first case, and that refinement is needed in patient selection for surgery at the MDT level.

Selection of appropriate surgical candidates who will benefit from aggressive therapy is critical to avoid subjecting vulnerable patients to invasive procedures with associated risks and potentially limited benefit. Several tools have been introduced to aid the clinician in making this decision. The ds-GPA is a well-validated prediction score designed to stratify patients into groups with a more or less favorable outcome.^20^ However, the ds-GPA does not select specifically for different treatment options, and it is, therefore, unclear to what extent it can be used to specifically identify patients who would benefit from surgical resection.

A number of studies have sought to assess whether the GPA can be used to guide the appropriateness of an operation.^30–33^ These studies have retrospectively analyzed operated cohorts of BM patients with the aim of identifying whether the GPA is able to robustly guide prognosis. Nieder et al.^33^ demonstrated that good performance status and good primary tumor control—2 important tenets of the GPA—were significantly associated with improved overall survival. Broadly, the GPA index correlated to survival, but there was wide variation between survival in different cohorts, and its predictive value was limited in patients with particularly short survival times. However, this study only included single BMs and would, therefore, likely exclude patients with some of the worst prognostic features.

This is echoed by Jakola et al.,^32^ who found no association between GPA and early mortality. This study included all new diagnoses of operated BMs in an unselected consecutive population and, therefore, more closely reflects its practical applicability at an MDT level which also operates in this way. The GPA did not predict for early mortality at 3 months or for adverse surgical outcomes.

All of these studies use the original Graded Prognostic Assessment. The ds-GPA introduces diagnosis-specific molecular and histological markers. Winther et al. assessed its prognostic value in operated BM patients over a 7-year period.^30^ Again, the GPA was unable to accurately predict “actual survival” especially in patients in whom survival was short. In addition, they also identified limitations in the real-world use of this more comprehensive tool, as the information required to calculate a ds-GPA was available less than 50% of the time, reflecting our own experience where there was frequently insufficient information available retrospectively in clinic letters and referral proformas.

In our study, there was no significant predictive value between “actual survival” and “predicted survival,” even when performing subgroup analysis by tumor type and by survival time. When considering patients who had a survival time of less than 6 months, there was no statistically significant concordance between actual and predicted survival. These results are significant as our main aim was to assess whether the ds-GPA was able to identify surgical patients with a poor predicted outcome who might not be considered for aggressive intervention.

This study also identified a shortcoming in the amount of information available to the MDT. There was frequently insufficient data available to accurately calculate a GPA (51% of exclusions, *n* = 64, see Table 1). Winther et al. reported similar difficulties, showing the ds-GPA could be applied to less than half of patients either because information was not always available or because the ds-GPA lacks an “Unknown” option and, therefore, cannot provide a score for patients in whom this data are in any way incomplete.^30^ Whole genome sequencing and identification of molecular variants for precision medicine are becoming embedded in neuro-oncology and are informing diagnosis, treatment stratification, and prognosis. Many neuro-oncology MDTs in the UK see large numbers of patients discussed in a relatively brief period. Regular review of referral proformas to ensure that they are clear and well-structured, and prompt revision of relevant referral information will improve workflow and efficiency within the MDT, and facilitate tailored decision-making.

## Limitations

Neurosurgeons typically meet a selected cohort of BM patients. Those patients submitted to the MDT for consideration of surgery will have already been selected to some degree for age, performance status, tumor size, tumor location, and tumor control. They are typically younger and fitter patients who have oligometastatic disease in locations that are amenable for resection. The GPAs were validated using a much wider cohort of patients who underwent many different types of treatment, and this may explain why it cannot be directly applied to surgical patients in the same fashion. In particular, it struggles to identify patients with very limited prognosis and is, therefore, not in isolation a robust measure to select or preclude patients from specific treatment strategies. Furthermore, decision-making which is based solely on expected survival also fails to recognize those patients in which surgery aims to provide symptomatic and functional improvement instead. Patients with raised ICP or large mass lesions causing symptoms via mass effect and compression may benefit from surgery in terms of functional improvement even if their survival is not prolonged.^34^

The GPA score was calculated contemporaneously at time of data review. For future work, it would be useful to collect this granular dataset, coupled with extended data such as frailty scores and body mass index score. This may identify other important independent predictors for prognosis that ds-GPA does not capture in order to improve the validity of the score.

## Conclusion

The ds-GPA is a useful tool for stratifying outcomes in brain metastases, but when applied in clinical practice at an MDT level, it was not able to reliably identify surgical BM patients who were likely to have a poor outcome. Its application in a challenging “real life” scenario also suffers from the limitations of incomplete information. Patient selection for aggressive therapies is crucial, and this study emphasizes the need for improved tools for relevant information to be provided to MDT and for treatment decisions to be individualized based on both patient and clinical cancer characteristics.
